# Changes in the Risk Perception of Food Safety between 2004 and 2018

**DOI:** 10.14252/foodsafetyfscj.D-20-00015

**Published:** 2020-11-20

**Authors:** Aiko Abe, Kazuo Koyama, Chie Uehara, Azusa Hirakawa, Itsuko Horiguchi

**Affiliations:** 1 Food Safety Commission Secretariat, Cabinet Office, Government of Japan, Akasaka 5-2-20, Minato-ku, Tokyo 107-6122, Japan; 2 The Support Center for Clinical Pharmacy Education and Research, Tokyo University of Science, 1-3 Kagurazaka, Shinjuku-ku, Tokyo 162-8601, Japan

**Keywords:** risk communication, risk management, risk perception

## Abstract

To afford the future agenda of risk communication through an evaluation of the past, we examined the changes in risk perception in the food safety sector over the 15 years (2004–2018) since the establishment of the Food Safety Commission of Japan (FSCJ) in 2003 by analyzing the data of the food safety monitor survey. Hazards such as contaminants including cadmium, methylmercury and arsenic, and pesticide residues caused high levels of concern among the public in 2004. In contrast, hazards such as food poisoning by harmful microorganisms and so-called “Health foods” have been ranked high among concerns since 2008 and 2014, respectively. Scoring of concern levels showed that concern related to food additives and pesticide residues intentionally added to foods and controlled has gradually decreased in a time-dependent manner. These concern scores were considerably lower in male monitors than in female ones; the scores were also lower for individuals with professional experience in the food sector than without the experience. The concern scores for contaminants were lower for males with professional experience. The concern scores related to food poisoning and health foods were not decreased and were remained high in recent years. These scores did not show clear dependence on job experience or gender of the monitors. A gap between food specialists and other attributes in the basic recognition of risk seems to make it difficult to communicate effectively and constructively among various interested individuals. To improve the quality of risk communication in the food safety field, it will be necessary to provide scientific knowledge and information regarding food safety management mechanisms for individuals without professional experience in the food sector, taking into account the changes in information media and influence on risk perception.

## 1. Introduction

According to Slovic et al^1^^)^, risk perception studies have aided risk analysis and societal decision-making by (i) improving methods for eliciting opinions about risk, (ii) providing a basis for understanding and anticipating public responses to hazards, and (iii) improving the communication of risk information among the general public, technical experts, and policy makers.

In Japan, there have been several studies carried out on risk perception in the food sector. Niiyama et al^2^^,^^3^^)^ reported on factors affecting risk perception and risk perception structure about hazards. The factors affecting risk perception structure include those derived from the characteristics of the risk or hazard (severity of health damage, accumulation or delayed effects in the body, controllability or avoidability, and benefits), personal factors (knowledge and image recall), and social factors (information exposure, regulatory measures, and trust in experts). Furthermore, Fujii et al^4^^)^ investigated the changes in risk perception before and after negative events related to nuclear power plants and showed that the occurrence of negative events and its reporting affect risk perception by decreasing trust and increasing fear perception. The study also showed that an administrator’s response could avoided a decline in trust after a negative event only if the administrator was assessed to be honest. Various other points, such as the relationship between risk perception and scientific literacy, have been investigated^5^^,^^6^^,^^7^^)^.

In 2003, the Food Safety Commission of Japan (FSCJ) was established by the enactment of the Food Safety Basic Law and the restructure of the food safety administrative system. The mission of the FSCJ is implementation of science-based risk assessment of food. Risk communication based on the results of risk assessments and scientific findings on food safety is another important mission of the FSCJ. Following these missions, the FSCJ has implemented various activities to communicate basic knowledge on food safety to the public as well as its risk assessment activities. Analysis of risk perception change following past risk communication activity in the field of food safety can provide a basis for examining the effects of previous efforts in risk communication and future directions. However, no study to evaluate the secular change in risk perception regarding food safety has been conducted in Japan. This study was, therefore, carried out to explore the changes in risk perception over time using the food safety monitor survey results published by the FSCJ to consider the measures required for future risk communication.

## 2. Methods

### Food safety monitor survey

The data from the food safety monitor survey conducted annually by the Food Safety Commission Secretariat (Secretariat) were used for this study. The Food Safety Monitor (monitor) is designed by the Secretariat to ask 470 individuals annually about their opinions on food safety in their daily life. These individuals, who have some knowledge or practical experience with food, sign a term of service, which currently lasts for 1 year and can be renewed for up to 5 years. This survey has been conducted every year since 2003, and the survey on the degree of concern about hazards has been conducted in the same format since 2004. The data of 15 years of research from 2004 to 2018, have been published on the FSCJ website^8^^)^. We compiled the survey data from 2004 (first survey) to 2018, along with the data of the 2011 survey conducted soon after the Great East Japan Earthquake, and analyzed those using statistical methods. The data have been anonymized by the Secretariat.

### Analysis based on job experience

Job experience was divided into the following two groups: “Food specialists” and “Others”. Monitors had qualifications or academic backgrounds related to food, but not necessarily professional experience. The job experiences of participants in the original survey each year were classified as follows: (i) “Food-related job” as job experience in food production, processing, distribution, and sales, or administrative experience in food safety; (ii) “Researcher” as professional job experience related to food research at testing and research institutes or universities; (iii) “Medical or educational positions”; and (iv) “Others”. Preliminary analysis showed similar trends for (i) and (ii), and for (iii) and (iv); thus, professional experience was divided into the following two groups: “Food specialists ((i) and (ii))” and “Others ((iii) and (iv))”. Individuals whose job experience was unknown were also assigned as “Others”.

### Questions to monitors in the survey and scoring concern levels

This survey asked monitors about hazards related to food safety; such as food additives, pesticide residues, veterinary antibiotics-induced antimicrobial resistant bacteria, contaminants (e.g., cadmium, methylmercury, and arsenic), food poisoning due to harmful microorganisms, and chemical substances eluted from food contact materials. The question was “What do you think of each hazard from the viewpoint of food safety?” To score the concern level about each hazard, the respondents assigned 0 points for “I do not know about the hazard” and “I am not concerned at all,” 1 point for “I am hardly concerned,” 2 points for “I am not certain,” 3 points for “I am somewhat concerned,” and 4 points for “I am very concerned”.

### Calculation of average concern scores

Average concern scores were obtained by dividing the sum of the points of concern levels about each hazard by the total number of monitors in each category of year, job experience, and/or gender. Differences in average concern scores between the populations were confirmed by *t*-test in Microsoft Office Excel.

### Others

We used the results of the survey conducted by the government. This study was approved in 2019 by the Ethical Review Board of Life Science Promoting Association, a public interest incorporated foundation (approval No. E2020-1).

## 3. Results

### Collected reply numbers classified by gender and job experience

The collected reply numbers in the food safety surveys of 2004, 2011, and 2018 are shown in Table 1. Reply samples classified food safety monitors according to gender and job experience were shown for each year. In our analysis, samples with missing values for one or more items in each study year were excluded from the analysis, and thus there are differences in numbers from 2004 and 2011 compared with those available on the FSCJ website. The monitor’s knowledge and experiences with food are assumed to be similar, although some variations are observed on their terms of service.

**Table 1.** Collected reply numbers classified by year, gender and job experience

	**Male**			**Female**		
	**2004**	**2011**	**2018**	**2004**	**2011**	**2018**
Food specialists	96	126	141	85	74	72
Others	29	29	45	225	152	90
Total	125	155	186	310	226	162

### Ranking order of concern level about food safety-related hazards

The ratio of monitors who answered “Very concerned” or “Somewhat concerned” about each hazard to the first question explained in the Methods is shown, in a descending order over the 15 years (Table 2). In the survey of 2004, contaminants including cadmium, methylmercury and arsenic and pesticide residues were ranked 1st and 2nd, respectively, among hazards; however, their ranks have gradually gone down since 2008, and they have been ranked lower than 5th place since 2016. In the survey of 2011, the hazard category ranked first was radioactive materials; however, it has continuously moved down and has been ranked lower than 5th place since 2017. In contrast, food poisoning due to harmful microorganisms was ranked 4th in 2004 and has been ranked 1st since 2008 except in 2011. Health foods was ranked low before 2013 but was ranked 2nd or 3rd from 2014 to 2018. Food additives was ranked 5th in 2004, 2007, and 2010.

**Table 2.** Ranking of food safety-related hazards causing concern (in descending order of percentage of " Very concerned" or "Somewhat concerned")

	**1st**	**2nd**	**3rd**	**4th**	**5th**
**2018**	Food poisoning	Antimicrobial resistance	Health foods	Mycotoxin	Allergen
**2017**	Food poisoning	Health foods	Mycotoxin	Antimicrobial resistance	Allergen
**2016**	Food poisoning	Health foods	Mycotoxin	Antimicrobial resistance	Radioactive materials
**2015**	Food poisoning	Health foods	Radioactive materials	Contaminants	Antimicrobial resistance
**2014**	Food poisoning	Radioactive materials	Health foods	Pesticide residues	Antimicrobial resistance
**2013**	Food poisoning	Radioactive materials	Contaminants	Health foods	Pesticide residues
**2012**	Food poisoning	Radioactive materials	Contaminants	Pesticide residues	Antimicrobial resistance
**2011**	Radioactive materials	Food poisoning	Pesticide residues	Contaminants	Antimicrobial resistance
**2010**	Food poisoning	Pesticide residues	Antimicrobial resistance	Contaminants	Food additives
**2009**	Food poisoning	Contaminants	Pesticide residues	Antimicrobial resistance	Food Contact materials
**2008**	Food poisoning	Contaminants	Pesticide residues	Antimicrobial resistance	Food Contact materials
**2007**	Contaminants	Pesticide residues	Food poisoning	Antimicrobial resistance	Food additives
**2006**	Contaminants	Pesticide residues	Food poisoning	Antimicrobial resistance	BSE
**2005**	Contaminants	Pesticide residues	Antimicrobial resistance	Food poisoning	Genetically modified Food
**2004**	Contaminants	Pesticide residues	Antimicrobial resistance	Food poisoning	Food additives

The data in this table are transcribed from the FSCJ website^8)^. All other tabulation results were independently obtained for this study, and they differ from those published by the FSCJ website. Two surveys were conducted in 2011, and the data from the first survey were used in this study.

The differences due to job experience in the order of hazards for which respondents answered “Very concerned” or “Somewhat concerned” for each hazard in 2004, 2011, and 2018 is shown in Table 3. In the surveys of 2011 and 2018, radioactive materials and food poisoning did not differ much depending on job experience. However, food additives and pesticide residues were ranked lower for food specialistss than for others in 2004 and 2011. In contrast, health foods were ranked higher for food specialistss than for others in 2011 and 2018.

**Table 3.** Difference by job experience: order of food safety-related hazards causing concern (in descending order of percentage of "Very concerned" or "Somewhat concerned")

		**1st**	**2nd**	**3rd**	**4th**	**5th**
**2018**	Food specialists	Food poisoning	Health foods	Antimicrobial resistance	Mycotoxin	Allergen
	Others	Food poisoning	Antimicrobial resistance	Contaminants	Mycotoxin	Health foods^1)^ Radioactive materials^1)^
**2011**	Food specialists	Radioactive materials	Food poisoning	Contaminants^2)^	Health foods^2)^	Pesticide residues
	Others	Radioactive materials	Food poisoning	Pesticide residues	Antimicrobial resistance^3)^	Food additives^3)^
**2004**	Food specialists	Contaminants	Food poisoning	Pesticide residues	Antimicrobial resistance	BSE
	Others	Pesticide residues	Contaminants	Antimicrobial resistance	Food additives	Genetically modified food

^1)^ 5th place with same rate.

^2)^ 3rd place with same rate.

^3)^ 4th place with same rate.

### Comparison of hazards by the average concern scores

 The average concern scores about food additives, pesticide residues, contaminants, radioactive materials, food poisoning, and health foods for the years 2004, 2011 and 2018 are compared with the changes in food-related hazards-induced concern scores by job experience and gender (Table 4). The average concern scores were lower for food additives than for pesticide residues and contaminants throughout the period. The concern scores for the three hazards generally decreased from 2004 to 2018 in a time-dependent manner. There was an apparent difference due to gender and job experience in the scores for food additives. The scores given for females were higher than those given for males, and the scores given for others were higher than those given for food specialists. The concern scores for pesticide residues and contaminants showed almost similar tendency in difference due to gender and job experience as those for food additives, although the difference was not distinct and was relatively small in the scores for contaminants. The concern scores for radioactive materials were the highest among the average concern scores for all food-related hazards of 2011, because the survey was carried out just after the nuclear accident in Fukushima. Thereafter, the scores for radioactive materials decreased significantly from 2011 to 2018. The scores for radioactive materials also showed similar tendency in difference due to gender and job experience as those for food additives, pesticide residues, and contaminants. In contrast, the concern scores for food poisoning and health foods showed a different tendency from those for the three hazards mentioned above. The scores for food poisoning and health foods in 2004 to 2018 did not indicate any obvious difference among the genders and job experiences.

**Table 4.** Differences of average concern scores about each hazards due to gender, job experience, and year

Hazards	Job experience		**2004**			**2011**			**2018**	
		**Male**	**Female**	*p* (gender)	**Male**	**Female**	*p* (gender)	**Male**	**Female**	*p* (gender)
**Food additives**	Food specialists	1.71	1.91		1.15	1.55	**	1.03	1.56	**
	Others	1.97	2.18		1.59	1.75		1.42	1.78	**
	*p* (job)		**		*	*		**		
**Pesticide residues**	Food specialists	2.23	2.34		1.41	1.66	*	1.18	1.68	**
	Others	2.31	2.48		1.69	1.81		1.60	1.76	
	*p* (job)							**		
**Contaminants**	Food specialists	2.47	2.65		1.52	1.66		1.42	1.90	**
	Others	2.76	2.56		1.69	1.77		1.91	1.86	
	*p* (job)	*						**		
**Radioactive materials**	Food specialists				2.10	2.41	**	1.33	1.76	**
	Others				2.41	2.48		1.67	1.99	*
	*p* (job)							*		
**Food poisoning**	Food specialists	2.36	2.35		1.90	1.88		2.06	2.25	
	Others	2.55	2.17	*	1.79	1.97		2.18	2.27	
	*p* (job)									
**Health foods**	Food specialists	1.88	1.82		1.42	1.73	*	1.90	1.69	
	Others	1.69	1.79		1.69	1.46		1.82	1.84	
	*p* (job)					*				

* and ** show significant difference in average concern scores between specialistss and others in the same years, and the same gender by *t*-test (*p* < 0.05 and *p* < 0.01, respectively).

## 4. Discussion

In the present study, we analyzed the data from the food safety monitor survey by the FSCJ to determine the annual changes in concern levels about food safety-related hazards and differences in those concern levels based on job experience and gender by (1) ranking the ratios of monitors feeling concern caused by the hazards and of (2) calculating the average concern scores for each hazard to compare the concern levels.

Concern levels about food additives, pesticide residues, and contaminants including cadmium, methylmercury and arsenic decreased from 2004 to 2011 in a time-dependent manner and continued to decrease or remained the similar levels from 2011 to 2018 (Tables 2 and4). The concern scores for food additives and pesticide residues were lower for food specialists than for others, and the time-dependent decrease was more distinct for males than for females (Table 4). Food additives used intentionally in foods are regulated by law and controlled during the manufacture process. Pesticide residues used in crops are also regulated by the law and controlled during the agricultural production. In food additives and pesticide residues, we considered that the progress in understanding of the hazards led to a reduction in the respective concern scores, especially for male food specialists. The concern scores for contaminants were also lower for male food specialists (Table 4). We considered that the reason was that the investigation results of contaminants had been published and the extent of the risk were understood especially by male food specialists. The concern scores for radioactive materials were the highest in 2011 and then decreased significantly from 2011 to 2018. In addition, the concern scores were lower for males than for females, and there was a tendency for the scores to be lower for food specialists than for others. This tendency was similar to those of food additives, pesticide residues, and contaminants in 2011 and 2018. The decrease in the concern scores from 2011 to 2018 may have resulted from better understanding of the contamination status of radioactive materials and the corresponding risk. In contrast, concern levels for food poisoning by harmful microorganisms and health foods did not show obvious time-dependent changes, and their ranks in terms of concern levels have remained between 1st and 4th every year since 2013. The time-dependent changes in the concern scores did not show clear dependence on job experience and gender (Tables 2 and 4). Although the number of food poisoning cases has decreased in the last 15 years, there were still 1,330 cases in 2018^9^^)^. The fact that harmful microorganisms cause actual health damage may be the reason why the high concern score has been maintained. In the case of health foods, new types of products have been continuously manufactured but a few illegal products may cause vague concern.

According to the food safety monitor survey conducted in fiscal year 2014^10^^)^, the most frequent response regarding why a monitor began to be less concerned about the hazard was, “It is because I learned the mechanism of risk assessment,” and the second most common reason was, “It is because risk management by the government is properly administrated”. The responses suggest that awareness of management practices affects risk perception. Moreover, the results in this study suggest that the recognition differs by attributes of monitors. Although a gender difference exists in risk perception tendency^11^^)^ in the field of food safety is known, this may apply only to the controlled hazards such as food additives and pesticide residues.

The results of this study were influenced by two aspects of social changes occurred in the 15 years, although the data are insufficient to understand the mechanistic causes of these phenomena. First is the enactment of the Food Safety Basic Law and restructuring of the administrative system for food safety, as described in the Introduction. During this period, risk communication has been actively conducted^12^^)^, and the mechanisms that ensure food safety have been recognized, particularly among specialists experienced in the food sector. This may contribute the significant decrease in the concern levels concerning controlled hazards such as food additives and pesticide residues. Given that several studies have shown that trust affects risk perception^4^^,^^11^^)^, this is an effect of not only understanding scientific content but also gaining trust through the understanding of systems and mechanisms. For people without job experience in the food sector, the concern levels for controlled hazards have decreased, but not to the same extent as that for food specialists. Therefore, non-food specialists should be considered as a priority target in future risk communication strategies. The second aspect is a change in media for food safety. Knowledge gaps between consumers and scientists raise consumer concerns, so accurate information of scientific background is required^13^^)^. The FSCJ is actively providing media with opportunities to explain their scientific reports. However, anyone can become an information sender through social media; therefore, the FSCJ needs to improve the measures of information transmission to individuals not previously covered.

In conclusion, the spread and delivery of information based on scientific knowledge should be considered to ensure food safety. A gap in the basic recognition of risks among various individuals depending on their attributes makes it difficult to communicate effectively and constructively. To improve the quality of risk communication in the food safety field, scientific knowledge and information on management mechanisms related to food safety should be provided to individuals without professional experience in the food sector in considering the changes in the influence on risk perception and information media.
